# Draft Genome Sequence of *Paenibacillus pini* JCM 16418^T^, Isolated from the Rhizosphere of Pine Tree

**DOI:** 10.1128/genomeA.00210-14

**Published:** 2014-03-20

**Authors:** Masahiro Yuki, Kenshiro Oshima, Wataru Suda, Yumi Oshida, Keiko Kitamura, Toshiya Iida, Masahira Hattori, Moriya Ohkuma

**Affiliations:** a Biomass Research Platform Team, RIKEN Biomass Engineering Program Cooperation Division, RIKEN Center for Sustainable Resource Science, Tsukuba, Ibaragi, Japan; b Center for Omics and Bioinformatics, Graduate School of Frontier Sciences, the University of Tokyo, Kashiwa, Chiba, Japan; c Japan Collection of Microorganisms/Microbe Division, RIKEN BioResource Center, Tsukuba, Ibaragi, Japan

## Abstract

*Paenibacillus pini* strain JCM 16418^T^ is a cellulolytic bacterium isolated from the rhizosphere of pine trees. Here, we report the draft genome sequence of this strain. This genome information will be useful for studies of rhizosphere bacteria.

## GENOME ANNOUNCEMENT

The rhizosphere, the zone of the soil that is adjacent to the plant roots, is a habitat of various species of bacteria, fungi, oomycetes, viruses, and archaea (1). Rhizosphere microbiota affect plant growth by nutrient mobilization or nitrogen fixation (1, 2). *Paenibacillus pini* strain S22^T^ (available from Japan Collection of Microorganisms as JCM 16418^T^) was isolated from the rhizosphere of pine trees (*Pinus densiflora*). Phylogenetic analysis based on the 16S rRNA gene sequence indicated that strain S22 represents a distinct lineage in the genus *Paenibacillus*, and it was designated the type strain of a novel species, *P. pini* (3). The cells of the strain are motile, spore-forming, Gram-positive, and rod-shaped, and the strain is strictly aerobic and able to hydrolyze starch and carboxymethylcellulose.

The genome of *P. pini* JCM 16418^T^ was sequenced using an Ion Torrent PGM system. The sequence reads of 585,913 were assembled using Newbler version 2.8 (Roche) into 68 contigs, with an *N*_50_ length of 258,739 bp. The assembly resulted in a draft genome sequence of 4,961,801 bp, with 26.4× redundancy and a G+C content of 42.0%. A total of 4,847 protein-coding genes and 81 RNA-coding sequences were detected after manual inspection of the annotations using the RAST server (4).

RAST annotations and the following analyses with the CAZy database (5) revealed that *P. pini* JCM 16418^T^ has several genes encoding α- and β-amylases classified in the glycoside hydrolase (GH) 13 and GH14 families, which supports the ability of this strain to hydrolyze starch. In addition, *P. pini* JCM 16418^T^ had genes encoding β-glucosidases of GH3, α- and β-galactosidases of GH2, GH4, GH35, and GH42, α-mannosidases of GH38, and chitinases of GH18. No typical homologous sequence with known cellulases was detected in the genome, and further investigation is necessary for understanding the cellulolytic system of this species. Nevertheless, the genome information will be useful for studies of rhizosphere microbiota.

### Nucleotide sequence accession numbers.

The genome sequence of *P. pini* JCM 16418^T^ has been deposited at DDBJ/EMBL/GenBank under accession no. BAVZ01000001 to BAVZ01000068.
